# AI-Driven Multi-Omics Approaches for Deciphering Small-Cell Lung Cancer Heterogeneity Using Nucleic-Acid-Based Features

**DOI:** 10.3390/cancers18193159

**Published:** 2026-09-29

**Authors:** Saman Zeeshan, Caila McVey, Shafaq Majeed, Zeeshan Ahmed, Mark Nichols

**Affiliations:** 1 Department of Biomedical and Health Informatics, School of Medicine, The University of Missouri-Kansas City, Kansas City, MO 64110, USA; 2 Department of Bio Sciences, COMSATS University, Islamabad 45550, Pakistan; 3 Rutgers Institute for Health, Health Care Policy and Aging Research, Rutgers, The State University of New Jersey, New Brunswick, NJ 08901, USA; 4 Department of Medicine, Robert Wood Johnson Medical School, Rutgers Health, New Brunswick, NJ 08903, USA

**Keywords:** small-cell lung cancer, AI, machine learning, multi-omics, tumor heterogeneity, nucleic acid profiling, resistance mechanisms

## Abstract

Background: Small-cell lung cancer (SCLC) is a rare but highly aggressive malignancy defined by rapid progression, early metastasis, and profound therapeutic resistance. Its extensive heterogeneity across genomic, transcriptomic, epigenetic, proteomic, and microenvironmental levels continues to undermine precision medicine. Emerging evidence highlights the complex role of epidermal growth factor receptor (*EGFR*) alterations, histologic transformation from *EGFR*-mutant non-small cell lung cancer (NSCLC) to SCLC, and the influence of distinct transcriptional subtypes on clinical behavior. Methods: This review synthesizes current literature encompassing nucleic-acid-based determinants of resistance, SCLC molecular architecture, transformation biology, and subtype evolution, integrating findings from genomic, transcriptomic, epigenetic, proteomic, imaging, and clinical studies. We additionally evaluated artificial intelligence (AI) and machine learning (ML) methodologies with potential to unify multi-omics, radiomics, and clinical variables to model subtype plasticity, clonal evolution, and therapy-induced rewiring. Results: The evidence demonstrates that traditional single-layer analyses are insufficient to capture the multiscale architecture of SCLC biology or the nonlinear interactions driving resistance, whereas AI-enabled multi-omics approaches offer advantages in enhancing subtype classification, supporting early detection, and improving prediction of therapeutic response. Key barriers such as tissue scarcity, limited biomarker availability, and extreme intratumoral heterogeneity remain significant challenges, though increasingly addressable through integrative computational frameworks. Conclusion: AI-driven systems-level integration reframes SCLC as a dynamically evolving, multilayered disease process, where nucleic-acid alterations are central to subtype evolution and treatment resistance, providing a conceptual model to guide biomarker discovery and therapeutic development. By leveraging AI-enabled multi-omics and radiomics, this framework has the potential to advance personalized clinical management and overcome longstanding diagnostic and therapeutic challenges in SCLC.

## Simple Summary

The extensive heterogeneity of small-cell lung cancer (SCLC) across genomic, transcriptomic, epigenetic, proteomic, and microenvironmental levels has continued to undermine precision medicine. This review aims to help understand SCLC biology, resistance, and therapy by utilizing current literature encompassing nucleic-acid-based determinants of resistance, SCLC molecular architecture, transformation biology, and subtype evolution, integrating findings from genomic, transcriptomic, epigenetic, proteomic, imaging, and clinical studies. By leveraging AI-enabled multi-omics and radiomics, this framework has the potential to advance personalized clinical management and overcome longstanding diagnostic and therapeutic challenges in SCLC.

## 1. Introduction: Reframing Small-Cell Lung Cancer Through an AI-Centered Lens

Small-cell lung cancer (SCLC) is an exceedingly rare, yet highly aggressive form of lung cancer characterized by rapid progression, early metastasis, and high rates of treatment resistance [1,2,3,4]. SCLC accounts for approximately 15% of all lung cancers and is associated with an especially poor prognosis, with a 5-year survival rate of only ~5%. At diagnosis, 70–80% of patients present with extensive-stage disease, reflecting the cancer’s rapid dissemination [5]. Moreover, the National Lung Screening Trial (NLST), with a multi-center effort of 33 U.S. medical centers and 53,454 people, reported that even among 125 cases detected early, 86% were already advanced, and early identification did not improve survival, underscoring diagnostic challenges [6]. While chemotherapy, radiation, and immunotherapy often produce initial responses, relapse is almost inevitable due to complex and dynamic resistance mechanisms.

Untreated SCLC carries a median survival of only 2–4 months due to its rapid progression [1], emphasizing the urgency of improving early detection and therapeutic forecasting. This aggressive behavior, coupled with high intratumoral heterogeneity, makes SCLC a particularly challenging malignancy to study and treat. The disease is deeply heterogeneous histologically, genetically, epigenetically, and phenotypically, indicating that resistance emerges through diverse evolutionary pathways.

One emerging area of complexity is the role of epidermal growth factor receptor (*EGFR)* alterations in SCLC. Although tyrosine kinase inhibitors (TKIs) have transformed the treatment landscape for *EGFR*-mutant non-small cell lung cancer (NSCLC), their benefit in SCLC remains unclear as TKIs are rarely used as a first-line standard treatment option for SCLC because of inconsistent clinical outcomes [7,8,9,10]. Patients harboring activating *EGFR* alterations relapse after a median progression-free survival (PFS) of ~12 months, largely due to acquired resistance [11]. This short-lived efficacy highlights a critical gap in understanding the mechanisms that underlie resistance in SCLC compared to NSCLC. In NSCLC, *EGFR* mutations account for 50–60% of resistant tumors, while *MET* and *HER2* amplification represent an additional 15–20%. Among these tumor mutations, 14% transformed to SCLC [12,13,14,15], and they only account for 5% of all lung cancers [16]. These findings underscore the need for further investigation into whether TKIs like *WEE1* inhibitors (Adavosertib), *CDK4/6* inhibitors (Trilaciclib), and PARP inhibitors (Olaparib) [7,8,9,10] for SCLC alone or in combination with chemoradiotherapy offer improved outcomes compared to standard-of-care regimens [4].

A more elusive mechanism of target-therapy resistance includes the histological transformation from *EGFR*-mutant adenocarcinoma to SCLC. Two different case studies involved repeat biopsies with patients treated with *EGFR* inhibitors. The first of these reported that 14% of the cases transformed into SCLC, while the second reported a less than 5% transformation [16,17]. This phenomenon arises under intense selective pressure from *EGFR* inhibitors and significantly worsens the prognosis of both limited- and extensive-stage SCLC. Notably, this change is not random; rather, it reflects the tumor’s intrinsic plasticity and adaptive potential under pharmacologic stress. It is further complicated by extensive intratumor heterogeneity and sex-specific differences in incidence, molecular biology, therapeutic response, and survival [18,19,20]. Transformation from NSCLC to SCLC has been found to occur as a result of treatment failure in 5–10% of patients and is most likely to occur between 16–49 months of the disease [21]. The molecular events driving this remain poorly defined [22], highlighting the urgent need for advanced, integrative methods to differentiate transformed SCLC from de novo form and uncover the sequence of events leading to transformation and therapy failure.

Traditional single-layer approaches like genomics alone, transcriptomics alone, or isolated clinical profiling cannot capture the multiscale architecture of SCLC biology. As SCLC is both biologically and clinically systemic, the disease should be conceptualized not as a linear tumor process but as a dynamic ecosystem shaped by tumor–immune interactions, neuroendocrine plasticity, and organ-level crosstalk [23,24,25,26,27,28]. Such a systemic perspective emphasizes that SCLC is not merely a collection of aberrant cells but a highly adaptive and evolving network of cellular and molecular interactions. This review emphasizes the importance of integrated, multi-omics, artificial intelligence (AI)-enabled analytics to reveal rare mechanisms, atypical mutations, and clinically meaningful signals overlooked by conventional methods.

AI and machine learning (AI/ML) offer powerful approaches to integrate radiomic, genomic, and clinical datasets, enabling enhanced prognostication and prediction of treatment response in SCLC. Numerous studies demonstrate that radiomics combined with AI can improve survival prediction, risk stratification, response assessment, and subtype differentiation in both limited- and extensive-stage disease [29,30,31,32,33]. There have been immense strides taken to improve medical research, diagnosis, and treatment using AI. For example, the AlphaFold AI model has improved the prediction of various protein structures with accuracy [34]. A second advancement is what is known as large language models, such as ChatGPT. These models can provide accurate diagnosis and treatment, as well as giving patients healthcare and disease prevention advice [35]. By leveraging high-dimensional datasets, AI provides insights into tumor behavior that were previously invisible, offering the potential to anticipate resistance before it manifests clinically. These findings support the relevance of AI-centered analysis in understanding SCLC’s biological heterogeneity and treatment resistance.

This review synthesizes current literature from NIH PubMed library of biomedical literature from 2016–2026 encompassing nucleic-acid-based determinants of resistance, SCLC molecular architecture, transformation biology, and subtype evolution. The literature-search methodology involved terms related to SCLC, AI/ML, multi-omics, genomics, transcriptomics, epigenomics, nucleic acids, tumor heterogeneity, molecular subtypes, plasticity, treatment resistance, neuroendocrine biology, prognosis, survival, and biomarkers. We included relevant original studies, reviews, and methodological papers addressing these themes in SCLC, while excluding studies unrelated to SCLC or the scope of this review. Reference lists of relevant publications were also screened to identify additional studies. The analysis integrates findings from genomic, transcriptomic, epigenetic, and proteomic studies, as well as imaging and clinical datasets. We additionally evaluated AI/ML methodologies with potential to unify multi-omics, radiomics, and clinical variables to model subtype plasticity, clonal evolution, and therapy-induced rewiring.

## 2. SCLC as a Systemic, Multi-Layered Disease

### 2.1. Interconnected Pathophysiology

SCLC is an inherently systemic malignancy due to early metastasis, neuroendocrine expression (NE), and near-universal inactivation of *RB1* and *TP53* [23]. These processes span molecular, immune, and microenvironmental domains, supporting a shift toward whole-system modeling rather than gene-centric approaches. In particular, the combined loss of *RB1* and *TP53* disrupts lineage fidelity and promotes epigenetic reprogramming, which underlies the remarkable cellular plasticity of SCLC and contributes to its ability to evolve under therapeutic pressure. This plasticity is most clearly evidenced by histologic transformation from NSCLC to SCLC, wherein tumor cells transition from an adenocarcinoma phenotype to a neuroendocrine SCLC-like state, particularly in the context of *EGFR*-targeted therapy. Recognizing SCLC as a multi-layered systemic disease enables better prediction of metastatic spread, therapy resistance, and subtype evolution. Although early transformation signals may exist, routine biomarker screening remains rare, representing a major unmet clinical need [24]. Advanced radiomic models can help differentiate SCLC from other lung lesions and non-invasively reflect systemic tumor biology, including immune microenvironment and neuroendocrine characteristics, further supporting whole-system modeling. An analysis of 103 studies was done by utilizing computed tomography (CT) scans and splitting them into non-contrast and contrast-enhanced scans. Machine learning (ML) classification models then generated radiomic models, concluding that these models can differentiate SCLC from other lung lesions [36,37]. Clinically, histologic transformation represents a key mechanism of acquired drug resistance through activation of lineage-defining transcriptional programs and suppression of *EGFR* dependence, underscoring the need for longitudinal molecular monitoring and repeat biopsy to detect phenotypic evolution.

### 2.2. Neuroendocrine Biology and Omic Convergence

Neuroendocrine differentiation, which is central to all SCLC subtypes [25], indicates shared pathways suitable for early diagnosis. Integrative omics is necessary to delineate subtype-specific molecular programs and track evolving tumor states [26,27,28]. By combining radiomics, transcriptomics, and proteomics, AI models can capture the neuroendocrine continuum, revealing subtle shifts that may precede clinical progression. Using extracted radiomic features from intra- and peritumoral regions results in the idea that integrating peritumoral radiomic and clinical data into a nomogram can non-invasively diagnose and plan surgeries for SCLC [38]. AI-based radiomics has been shown to differentiate SCLC subtypes and distinguish SCLC from NSCLC using CT imaging [38,39], indicating its potential to detect neuroendocrine differentiation patterns.

### 2.3. Multi-Omics Mapping of Mutation Burden and DNA-Level Features

SCLC displays one of the highest mutation burdens among solid tumors, with an average nonsynonymous mutation rate of 5.5 per megabase and prominent G-to-T transversions characteristic of tobacco mutagenesis [40]. Whole-genome analyses report >50,000 somatic mutations per tumor, including extensive missense, nonsense, splice-site, and indel variants, with near-universal inactivation of *TP53* and *RB1* driving genomic instability notably in kinases, G protein-coupled receptors (GPCRs), and chromatin modifiers, ~53% of which are predicted to affect function [40,41].

At the protein level, dysregulation of deoxyribonucleic acid (DNA) repair pathways (*PARP1*, *CHK1*) and chromatin regulators (*EZH2*) provides both therapeutic opportunities and mechanistic insight into resistance. DNA repair proteins are frequently overexpressed independent of gene amplification, supporting PARP inhibitor evaluation (olaparib, rucaparib, talazoparib) in ongoing trials [1,42]. Less frequent but pathogenic alterations in *KEAP1* (~3%), *TET2* (~2%), *SMARCA4* (~1.5%), and *ZNF703* (2.9%) further implicate oxidative stress, epigenetic, and transcriptional pathways in SCLC biology [43,44].

Proteomic and phosphoproteomic studies highlight *CHK1*, *ATR*, *ATM*, *CDK2*, and *GSK3A* as key regulators and identify *HMGB3* (poor prognosis) and *CASP10* (favorable) as prognostic markers [45]. Immune profiling demonstrates many SCLC tumors are immunologically “cold,” whereas “hot tumors” express higher T-cell, MHC, and checkpoint signatures and may better respond to immunotherapy; *ZFHX3* mutations correlate with improved chemoimmunotherapy responses [46,47]. AI can integrate these multi-layered datasets to identify correlations between genomic instability, neuroendocrine plasticity, and immune phenotype, guiding personalized therapy.

## 3. Nucleic-Acid-Based Determinants of Transformation and Resistance

Gene alterations appear to induce resistance to targeted therapy and are responsible for a large fraction of cancer recurrences. The histological transformation of *EGFR*-mutant NSCLC to SCLC is a vivid example of molecular complexity and tumor plasticity, where the genetic identity remains partially preserved, but cellular phenotype dramatically shifts to evade therapy. The involvement of genomic drivers in this transformation is a well-recognized mechanism of acquired resistance to chemotherapy, targeted therapy, immunotherapy, and radiotherapy, profoundly worsening prognosis.

Central to therapy resistance is tumor evolution under the selective pressure of *EGFR*-targeted therapy in *EGFR*-mutant NSCLC, which in some cases leads to histologic transformation into SCLC, in a median timeframe of 1.1 years, which reflects marked tumor plasticity and molecular complexity [15]. A key difference with de novo SCLC is that it starts as SCLC in the beginning and arises with no previous history of a different lung cancer. Patients with de novo SCLC also exhibit high initial response rates to standard therapies (60–70%); however, resistance rapidly develops and relapse is nearly universal. Drug-resistant mechanisms include intratumoral heterogeneity change, cancer-stem-cell-dependent mechanisms, apoptosis invasion, DNA repair, and tumor microenvironment (TME) alterations. A response factor to consider is that sex-specific differences have been identified across incidence, survival, and treatment response [19,20]. SCLC presents differences in sexes based on smoking habits in men and women. This disparity means that more women than men are detected to have SCLC. Additionally, SCLC exhibits significant intratumoral heterogeneity, making comprehensive targeting challenging [18]. The presence of multiple co-existing clones with distinct vulnerabilities complicates treatment design and underscores the need for predictive models that can anticipate clonal dominance.

The molecular events driving the transformation remain poorly understood [22], emphasizing the need for deep nucleic-acid-level resolution to map clonal architecture, selective subtype switching, and lineage plasticity. AI-enabled models can integrate multi-omics data to reconstruct evolutionary trajectories, identify early markers of transformation, and provide actionable insights for therapy personalization. These models predict early disease progression and survival by extracting specific tumor regions to personalize diagnosis and treatment. Certain parameters, such as smoking, sex, or demographics, influence treatment response, but to determine their effect on prognosis and treatment, univariate analysis can be done. Despite limitations of scan section thickness, risk factors (e.g., smoking), and cohort size, early PFS-prediction models [30,33] demonstrate that AI can detect subtle pre-resistant patterns, supporting its use in decoding nucleic-acid-driven transformation.

## 4. Tumor Heterogeneity Through Nucleic-Acid-Based Profiling

To date, no study has systematically characterized universal resistance in SCLC using integrative multi-omics, although such frameworks are essential for identifying actionable biomarkers and informing targeted therapies. Existing genomics and transcriptomics studies reveal only segments of SCLC biology. Without integration across multiple layers, critical interactions and rare but clinically significant mutations remain hidden, limiting our ability to anticipate resistance.

A multi-omics, nucleic-acid-centered analytic framework is required to uncover atypical and rare mutations, transformation-linked transcriptional reprogramming, convergent pathways driving universal resistance, and hidden molecular relationships invisible to single-layer analyses. Combining these data with AI-based radiomics further allows for spatial mapping of heterogeneity, enabling the detection of evolving clones across tumor regions. AI-enabled radiogenomic strategies can help overcome the limitations of isolated genomics or transcriptomics by detecting spatial and functional heterogeneity from imaging. Several AI models already combine radiomics with clinical variables to generate accurate survival or response predictions by extracting tumor regions to assist in personalizing diagnosis and treatment of the disease [29,32]. One study included 93 patients with extensive-stage SCLC treated with chemoimmunotherapy, from which 1781 radiomic features were extracted; the combined radiomic-clinical model achieved an average concordance index (C-index)/AUC of approximately 0.8487, indicating moderate predictive performance [32]. The second study analyzed 200 patients with limited-stage SCLC and 1037 extracted features, with a reported AUC of 0.72 and 0.71 in the training and test groups [29]. Although this is a good step in combining AI models with clinical variables, improvement to make the tests even more accurate must be done.

To improve these approaches, several steps are needed: (i) development of larger, multicenter datasets with standardized imaging protocols to enhance generalizability; (ii) rigorous external validation; and (iii) reduction of feature dimensionality through feature selection or regularization methods to mitigate overfitting. To address the challenge of small sample sizes and high-dimensional omics data, emerging machine learning strategies such as transfer learning, self-supervised learning, and multimodal data integration are particularly promising. Deep learning models such as Sybil illustrate how large-scale imaging data can be leveraged to learn robust representations without manual feature engineering, although their application to SCLC specifically remains to be established.

## 5. AI-Driven Multi-Omics Integration: A New Framework for Understanding SCLC

### 5.1. Limitations of Traditional Analytic Frameworks

An important issue with AI models is that their internal decision-making processes are unclear, making the transition from inputs to outputs difficult, causing skepticism from doctors and patients [48]. A more persistent challenge in SCLC research is the lack of systematic multi-omics studies investigating universal resistance mechanisms. Conventional approaches fail to model the nonlinear interactions across biological layers or the adaptive nature of tumors under treatment pressure. Consequently, they miss latent biomarkers and evolutionary transitions hidden in siloed datasets. This siloed approach may explain why clinical outcomes remain unpredictable despite advances in targeted therapy. AI/ML approaches applied to multi-omics research can reveal latent biomarker constellations obscured in single-omic analyses.

AI/ML approaches have the ability to reveal latent biomarker constellations and nonlinear interactions not identifiable through single-omic analyses. For instance, AI models can detect complex patterns in gene expression, mutation co-occurrence, and epigenetic modifications that predict therapeutic response or transformation risk. Radiomic AI models have demonstrated the ability to stratify prognosis and predict PFS/OS using CT imaging features capabilities far beyond traditional analytic methods [29,32,33]. A study using the XGBoost model, a gradient boosting algorithm, was conducted using patients diagnosed with SCLC between 2000–2018, divided randomly, and analyzed based on demographics, tumor specifics, and treatment. The results showed that the XGBoost maintained the highest level of precision in predicting early mortality, compared to other ML algorithms and traditional approaches. These models demonstrate strong predictive performance, achieving high accuracy in mortality prediction in SCLC chemotherapy patients, suggesting significant clinical utility [49]. A summary of the methods and tools used in SCLC is found in Table 1. Details are available in the Supplementary Table S1.

### 5.2. Multi-Agent AI as a Next-Generation Framework

A central hypothesis in next-generation SCLC research is that specialized AI agents, each modeling different biological scales, can collaboratively infer biomarkers and resistance pathways invisible to human interpretation, which is particularly relevant for deconvoluting SCLC’s plasticity, including transformation from NSCLC under *EGFR*-targeted therapy. Genomic sequencing confirming preservation of the original *EGFR* mutation after transformation supports a clonal relationship between pre- and post-SCLC states, suggesting a predictable evolutionary adaptation instead of a random event [4,15]. To evaluate genomics of the NSCLC-SCLC transformation, tumor cell heterogeneity has been explored through single-cell analysis, mapping the intratumoral immune cell landscape from transformed SCLC patients. Tumor immune microenvironment (TIME) analysis revealed enrichment of T cells and B cells in transformed SCLC, while lymphocytes were observed to promote differentiation in lung adenocarcinoma (LUAD) cells [55]. A second study demonstrated that the plasticity and heterogeneity of EGFR-mutant, transformed SCLC tumors are characterized by the presence of both epithelial neuroendocrine (NE) and mesenchymal non-NE cell populations [56]. Examinations of SCLC patients have also been done to identify distinct mutational profiles that had not previously been reported in cases of histologic transformation to SCLC [57]. This change in EGFR-mutant NSCLC suggests that lineage plasticity undermines targeted therapy by enabling tumors to evade oncogene dependence [58]. To address key limitations in understanding NSCLC-to-SCLC transformation, further research is needed to better define the timing and molecular characteristics of transformed SCLC, as well as to expand the number of patients and matched pre-treatment samples studied [25]. In addition, blood-based biomarkers may enable early detection of SCLC and, when followed by confirmatory CT imaging, could facilitate earlier diagnosis and potentially improve survival outcomes [59].

Moreover, multi-agent AI can integrate radiomic, genomic, and transcriptomic inputs to simulate tumor evolution, effectively predicting the trajectory of resistant clones before they dominate the tumor population. If there are conflicting results between imaging and genomic data, mechanisms such as transformers use self-attention mechanisms to weigh the importance of conflicting signals and prioritize the more important result [60]. AI applied to radiomics has already shown promise in detecting phenotype shifts and predicting therapeutic response in SCLC. Multi-institutional studies demonstrate that AI-derived risk scores can identify patients most likely to benefit from chemoimmunotherapy [31]. These models suggest that SCLC evolution may be computationally predictable when integrating high-dimensional imaging and molecular data. Deep-learning tools such as the Sybil model have demonstrated excellent predictive accuracy for lung cancer risk. A study assessing 15,000 NLST participants was conducted with the program being run on a radiology reading station and validated on three different datasets. This study concluded that Sybil could predict a person’s future cancer risk using one low-dose computed tomography (LDCT) scan, supporting their potential for SCLC risk stratification [53].

### 5.3. Explainable Artificial Intelligence and Trustworthy Multi-Omics Models in SCLC

Despite the remarkable predictive performance of AI in analyzing high-dimensional multi-omics datasets, its successful adoption in SCLC depends not only on predictive accuracy but also on the transparency, reproducibility, and clinical interpretability of its decisions [26]. Conventional deep learning models often operate as “black-box” systems, producing highly accurate classifications or treatment-response predictions without clearly explaining which biological features influenced the outcome. This limited interpretability remains a major barrier to clinical acceptance, particularly in SCLC, where treatment decisions are influenced by the complex interaction of genomic instability, neuroendocrine plasticity, epigenetic reprogramming, tumor microenvironment dynamics, and immune heterogeneity.

Explainable artificial intelligence (XAI) offers an important solution by revealing the molecular and clinical rationale underlying AI-generated predictions. Rather than providing only final classification, XAI techniques can quantify the contribution of genomic alterations, DNA methylation signatures, transcriptomic programs, radiomic characteristics, proteomic profiles, and clinical variables to model outputs. Such interpretability enables researchers and clinicians to understand how distinct molecular layers collectively influence tumor subtype classification, therapeutic response, and disease progression. Recent AI frameworks increasingly employ attention-based architectures and feature-attribution methods that identify the biological pathways and data modalities most responsible for prediction, thereby improving both model transparency and clinician confidence [26]. Specific feature-attribution approaches can provide more detailed insight into how individual features and cross-omics interactions contribute to model predictions. SHapley Additive exPlanations (SHAP), for example, can quantify the contribution of individual genomic, epigenomic, transcriptomic, proteomic, radiomic, and clinical features and can also be extended to examine interactions between features. Local Interpretable Model-Agnostic Explanations (LIME) can provide sample-specific explanations by identifying the features that most strongly influence an individual prediction, while integrated gradients can attribute predictions to input features in differentiable deep-learning models. In multi-omics applications, these approaches can help determine whether features from different molecular layers jointly contribute to outcomes, such as whether a genomic alteration, DNA-methylation pattern, and transcriptional program collectively influence treatment-response prediction. Attention-based approaches may provide complementary information regarding important features or modalities, although attention weights should not necessarily be interpreted as causal explanations.

For SCLC, XAI is particularly valuable because tumor evolution is driven by coordinated molecular alterations rather than isolated biomarkers. Integrative explainable models can uncover biologically meaningful relationships across multiple omics layers, for example, linking transcriptional subtype transitions with epigenetic alterations, radiomic phenotypes, and tumor microenvironment characteristics that collectively contribute to therapy resistance or histologic transformation. Feature-attribution analyses, including SHAP interaction analyses, can further determine whether combinations of features across different omics layers contribute disproportionately to a model prediction. For instance, an explainable model could reveal that a specific genomic alteration has a stronger association with treatment resistance when accompanied by a particular DNA-methylation pattern and transcriptional program. These systems-level insights extend beyond conventional statistical analyses, facilitating biomarker discovery while improving mechanistic understanding of disease progression and enabling more informed precision oncology strategies [61].

Structural bioinformatics frameworks can provide a complementary perspective by connecting AI-derived molecular predictions with mechanistic information at the protein and compound levels. For example, the DrLungker framework [62] integrates deep-learning and ensemble models with molecular descriptors and incorporates feature-level explainability and downstream structural analyses to investigate the molecular basis of predicted lung-cancer drug activity. Although DrLungker is not itself a multi-omics SCLC model, it illustrates how predictive modeling, explainability, and structural validation can be combined to move from computational predictions toward biologically interpretable mechanisms. Similar hierarchical strategies could be adapted to SCLC multi-omics models to connect predictive features across molecular layers with biological pathways, protein structures, molecular interactions, and therapeutic phenotypes.

Equally important, the clinical translation of AI-based multi-omics models requires rigorous validation and standardization. Models developed using single-center datasets frequently exhibit reduced performance when applied to external populations because of differences in sequencing platforms, imaging protocols, sample processing, and patient demographics. Future research should therefore prioritize multicenter validation, harmonized preprocessing pipelines, external benchmarking, prospective clinical evaluation, and transparent reporting standards. Integrating explainability with robust validation will improve model reliability, promote clinician trust, and accelerate the incorporation of AI-driven multi-omics into routine precision management of SCLC [63]. Importantly, XAI-derived feature importance should be interpreted as evidence of model reliance rather than direct evidence of biological causality. Combining attribution results with pathway analysis, experimental validation, and structural or mechanistic analyses will therefore be essential for translating computational associations into biologically and clinically meaningful insights.

### 5.4. Potential for Early Detection and Risk Stratification

AI-enabled genomic and imaging-based screening tools may detect transformation-prone tumors earlier and uncover rare pre-resistant molecular patterns. As existing biomarkers lack sensitivity and specificity for early detection in a profoundly heterogeneous disease, AI-based multi-biomarker approaches can support reliable staging, risk prediction, and therapeutic decision-making. Early identification of high-risk patients could facilitate proactive treatment adjustments, potentially delaying or preventing relapse. These multimodal biomarkers, especially CT radiomics, can detect early structural and microenvironmental changes associated with transformation or emerging resistance. Studies using contrast-enhanced CT radiomics have already predicted PFS and early progression and survival by extracting and analyzing radiomic features from specific tumor regions to personalize diagnosis and treatment [30,33]. Overfitting is a risk when handling high-dimensional imaging datasets; however, to reduce this risk, features are removed that have high correlations between two feature sets [33]. Another study on NLST data based on CT scans of 12–54% of the participants with SCLC detected them at stages III and IV. Because SCLC often presents at advanced stages despite screening efforts, AI-enhanced early detection is especially critical to overcome the diagnostic limitations illustrated by large-scale trials such as the NLST [6].

## 6. Molecular Subtypes and Transcriptional Lineages in SCLC

WHO classifies SCLC into pure and combined types [51]. Pure SCLC expresses neuroendocrine markers such as CD56, *ASCL1*, *NEUROD1*, and chromogranin A, whereas combined SCLC contains both SCLC and NSCLC components. In combined SCLC, non-small cell carcinoma components such as squamous cell carcinoma or large cell carcinoma may appear adjacent to classic SCLC regions, reflecting complex tumor architecture. Molecularly, SCLC subtypes are defined by *ASCL1* (SCLC-A), *NEUROD1* (SCLC-N), *POU2F3* (SCLC-P), and the immune-inflamed SCLC-I subtype lacking expression of the three transcription factors (TFs) [40,46,60]. *ASCL1* and *NEUROD1* regulate neuroendocrine programs (*INSM1, SYP, CHGA, NCAM1*) and oncogenes such as *MYCL1*, *MYC*, and *SOX2* [52,64,65,66,67,68]. SCLC-A may further subdivide into SCLC-A1 and SCLC-A2 depending on *NKX2-1* and *SOX1* expression [64]. SCLC-P represents a low-neuroendocrine, tuft-cell-like subtype dependent on *POU2F3* [23]. Tuft-cell biology underlies the SCLC-P subtype, where *POU2F3*-driven tumors express chemosensory programs that detect extracellular bioactive molecules and regulate immune responses [67], highlighting unique therapeutic vulnerabilities. The distribution of these subtypes is approximately SCLC-A 36%, SCLC-N 31%, SCLC-I 17%, and SCLC-P 16% [51].

Longitudinal tracking of SCLC during clinical treatment is important because of the evasive nature of cellular plasticity. Applying an AI classifier, a variant of XGBoost trained for each SCLC subtype (SCLC-A, N, P, and I), the classifier was able to accurately subtype tissue samples into the respective molecular subtypes with 96.6% accuracy that was cross-validated due to lack of plasma samples [52]. This method was adapted for plasma-based, non-invasive monitoring of subtype switching and for longitudinal tracking of cellular plasticity in SCLC. Applying these techniques to SCLC longitudinal tracking can improve treatment plans and help combat the resistant and evasive nature of SCLC.

**Figure 1:**
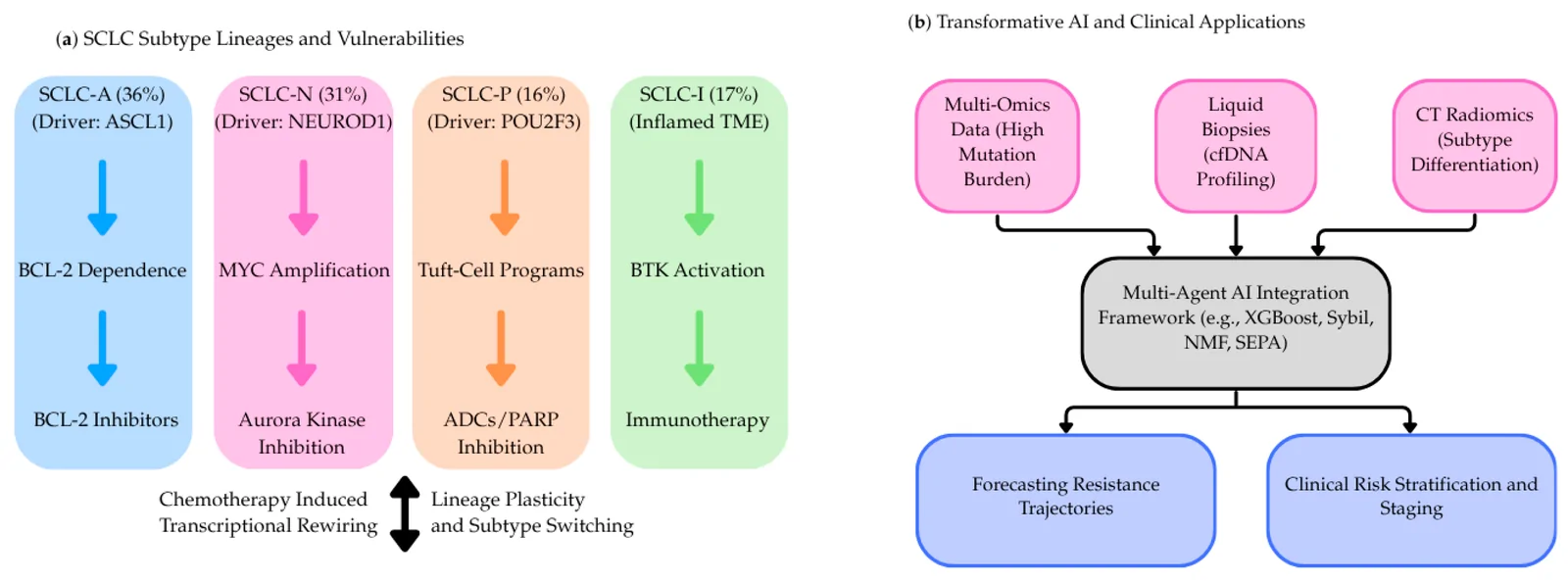
(**a**) SCLC subtype lineages and vulnerabilities. (**b**) Transformative AI and clinical applications. The pink boxes include multi-model data inputs, while the blue boxes include final outcomes.

Therapeutic vulnerabilities in SCLC are closely linked to subtype-specific transcriptional programs and lineage dependencies. SCLC-A tumors exhibit strong dependence on anti-apoptotic signaling pathways (e.g., to B-cell lymphoma 2 (*BCL2*)), making them more responsive to *BCL2* inhibitors, whereas SCLC-N tumors are characterized by enhanced proliferative and cell-cycle activity, conferring sensitivity to Aurora kinase inhibition. In contrast, SCLC-P tumors display non-neuroendocrine, tuft-cell-like features and metabolic dependencies that render them more responsive to anti-metabolite-based therapies and PARP inhibition [46]. The SCLC-I subtype, defined by an inflamed TME and reduced lineage-defining transcription factor expression, shows comparatively higher sensitivity to immune-modulatory approaches, including Bruton tyrosine kinase (*BTK*) pathway targeting and immunotherapy-based strategies [52]. Importantly, these subtype-specific vulnerabilities are not static, as transcriptional plasticity and therapy-induced lineage switching can reprogram therapeutic dependencies over time, complicating durable treatment stratification. SCLC subtype lineages and vulnerabilities, as well as AI and clinical applications, are presented in the diagrams in Figure 1.

Responsiveness to therapeutic intervention differs across SCLC subtypes and is shaped not only by intrinsic transcriptional programs but also by interactions with the TME and mechanisms of immune evasion. For example, SCLC-P is sensitive to anti-metabolites and PARP inhibitors, reflecting its distinct metabolic and DNA damage response dependencies, whereas SCLC-N demonstrates sensitivity to Aurora kinase inhibitors (AKIs) due to its proliferative phenotype. SCLC-A shows increased dependence on anti-apoptotic signaling and is therefore more responsive to *BCL2* inhibitors. In contrast, the SCLC-I subtype is characterized by an immune-inflamed TME and reduced neuroendocrine marker expression, making it comparatively more responsive to immune-modulatory approaches, including *BTK* inhibition [52]. These subtype-specific vulnerabilities highlight the importance of integrating both tumor-intrinsic and microenvironmental features when considering therapeutic strategies in SCLC [26,28,69].

Implementation of subtype-guided therapy does come with its limitations. In order to confirm histological subtyping, a tissue biopsy, which is expensive and invasive, must be performed. In addition, samples need to yield a sufficient amount of tissue, creating the possibility of a repeat biopsy [69]. Clinical implementation of subtyping also comes with its own challenges. The biggest includes a lack of a standardized classification system to translate subtype findings. Classification methods have been discovered, such as ribonucleic acid (RNA)-sequencing, methylation profiling, and immunohistochemistry; however, they each have resulted in subtype inconsistencies [70].

AI-assisted subtype detection using transcriptomics, radiomics, or computational pathology allows identification of early subtype-switching events, potentially before they are clinically apparent. These models can discern marginal shifts in gene-expression programs or radiologic patterns that indicate phenotypic drift long before it is clinically evident. With strong support from radiomics literature, AI-assisted subtype detection has demonstrated accurate discrimination of SCLC vs. NSCLC and subtype predictors of treatment benefit [36,38,39].

## 7. Treatment Implications and Subtype Plasticity

Recent molecular profiling challenges the historical view of SCLC as homogeneous, revealing substantial cellular plasticity influenced by environmental and therapeutic pressures [71,72]. Chemotherapy can drive *ASCL1*-high (SCLC-A) tumors to transition toward *NEUROD1*-driven (SCLC-N) or *YAP1*-expressing non-neuroendocrine states, contributing to relapse and resistance [40,47]. Each subtype exhibits unique vulnerabilities: *MYC*-amplified SCLC-N may respond to AKIs and epigenetic inhibitors; SCLC-I is more immunotherapy-responsive; and SCLC-P may benefit from antibody–drug conjugates (ADCs) targeting tuft-cell-related pathways [71]. Recognizing and anticipating these transitions is critical for designing adaptive therapeutic strategies that preempt resistance.

Early detection tools such as circulating-free DNA (cfDNA) methylation profiling remain under development [65]. One study, however, found that in six patients, tumor-specific methylation patterns were detected in SCLC cfDNA samples and that these levels of tumor methylation in cfDNA corresponded with survival outcomes [73]. A second, more recent study, included thirty-two extensive-stage SCLC patients and concluded that there is an association with increased concentrations of cfDNA and the risk of disease progression, making it useful in anticipating radiological detection of disease progression [74]. Another study focused on circulating-tumor DNA (ctDNA), a subset of cfDNA, that included 33 patients and concluded that with no clear guidance on using ctDNA clinically, it is not ready to be used in a clinical setting [75]. A targeted capturing panel was designed based on cfDNA to predict tumor TF activity to distinguish SCLC histology and infer its subtypes. This study found that out of three samples, all showed high levels of *NEUROD1* activity, supporting the potential of this approach being used to characterize genotype and phenotype links in SCLC [76]. Liquid biopsies are another option that can be used to quantify somatic variants in cfDNA. In this study, 140 plasma samples were used, and it resulted in disease-associated mutations in 85% of samples with mutant allele frequencies, as well as detecting alterations in 10 additional genes (*PTEN, NOTCH1-4, MYC, MYCL1, PIK3CA, KIT,* and *BRAF*). These findings support the conclusion that liquid biopsies can improve disease monitoring, depth of treatment response, and potentially warn of disease relapses [77]. One of the more unknown aspects of SCLC is prognostic values of cfDNA and circulating tumor cells. This study analyzed cfDNA in 46 SCLC patients during chemotherapy and circulating tumor cells in 21 patients before therapy. It was found that high cfDNA levels are associated with PFS and OS and that cfDNA levels at three weeks and at disease progression were associated with patients’ outcomes [78]. The last study applied whole genome next-generation sequencing to circulating cfDNA from 39 patients with limited-stage SCLC and 30 patients with extensive-stage SCLC. This resulted in identification of tumor-related changes in 94% patients with limited-stage SCLC and 100% patients with extensive-stage SCLC, supporting the idea that a cfDNA genome-wide copy number approach is a beneficial way of monitoring patients through treatment and identifying therapeutic targets in more than 50% of patients [50]. While promising, more data are still needed to support cfDNA methylation as an early detection tool.

Limited-stage SCLC is managed with early chemoradiation and prophylactic cranial irradiation when appropriate, while first-line therapy for extensive-stage disease consists of platinum–etoposide with atezolizumab, durvalumab, or carboplatin [79,80]. Targeted therapies focusing on *MYC*, *NOTCH*, *SOX2*, and other subtype-specific pathways, as well as emerging immunotherapy strategies using T-cell-inflamed gene signatures, are under investigation [40,81]. Food and Drug Administration (FDA)-approved therapies for relapsed extensive-stage SCLC include Tarlatamab-dlle (Imdelltra), a bispecific T-cell engager (BiTE) that works by binding to *DLL3* on tumor cells and CD3 on T cells, enabling the immune system to attack the cancer, and Lurbinectedin (Zepzelca), a selective inhibitor of oncogenic transcription, primarily inhibiting genes regulated by RNA polymerase II for metastatic SCLC [82]. Surgery plus adjuvant chemotherapy may benefit early-stage limited disease [59]. Therapy can induce transitions between *ASCL1*-, *NEUROD1*-, and *POU2F3*-driven states via transcriptional rewiring.

AI-driven models can integrate genomic, transcriptomic, and radiomic data to predict subtype-specific drug responses, early transformation events, and optimal therapeutic combinations, enabling truly personalized SCLC care. AI-based clustering techniques such as non-negative matrix factorization (NMF) have identified immune-enriched (“immune-hot”) SCLC subtypes that exhibit improved responsiveness to PD-1/PD-L1 immunotherapy [46]. Several studies predict responses to PD-1 inhibitors using radiomics plus clinical variables [83] and chemoimmunotherapy benefit using CT-derived risk scores [31].

## 8. Prognosis and Survival Factors

Prognosis in SCLC is strongly stage dependent. Limited-stage disease achieves long-term survival rates of 20–25%, whereas extensive-stage disease carries a median survival of only 8–13 months. Even within the same stage, outcomes can vary widely due to molecular, demographic, and treatment-related factors, emphasizing the importance of precision prognostication. Genomic features also influence outcomes: 4q12 amplifications (e.g., *KDR*, *PDGFRA*) correlate with better prognosis, while *CCNE1* and *APC* alterations and subtypes such as *STK11*-mutant or high-TMB SCLC are linked to more aggressive behavior [43].

Demographic factors contribute as well. The median age at diagnosis was 69 in 2019 [84], and females show slightly improved survival compared to males, potentially reflecting sex-based biological or immune differences, though these remain poorly understood [66]. Lifestyle factors, comorbidities, and treatment tolerability further modulate outcomes, particularly in older patients where aggressive therapy may be limited.

In recent years, machine learning survival models, such as DeepSurv, XGBoost, and Gradient Boosting Machine (GBM) have been used to predict 1–2-year survival in SCLC patients with greater accuracy than traditional Cox models. Treatment type, age, stage, and performance status were the most important variables used in these models to determine 1–2-year survival [85,86]. Utilizing XGBoost, Random Forest (RF), Multilayer Perception (MLP) and K_Nearest Neighbors (KNN) on SEER data from 2000–2018, a study predicted 90-day mortality after chemotherapy in SCLC patients [49].

AI-based survival modeling integrating genomics, demographics, imaging, and clinical course can enable more accurate prognostication and individualized treatment planning. It has been reported to improve prediction accuracy across several SCLC cohorts [29,30,87]. By incorporating multi-dimensional data, AI models can stratify patients into precise risk categories, predict likely relapse timing, and guide therapy intensification or de-escalation. These models help refine prognostic stratification beyond traditional staging.

## 9. Challenges in Studying Nucleic-Acid-Driven SCLC Biology

SCLC research faces longstanding barriers such as limited tumor tissue availability, extreme molecular complexity, rapid disease progression, intratumoral heterogeneity, and cellular plasticity [64,66,88,89]. Issues with intratumoral heterogeneity are largely due to the coexistence and subtype exclusion, which makes treatment challenging [90]. Small sample sizes and SCLC-specific datasets have also proven to be a big issue in SCLC research due to its low incidence in the population [39]. These factors collectively hinder the development of robust predictive biomarkers and the ability to validate therapeutic strategies systematically. Insufficient biomarkers for response and resistance, along with historically low research investment in SCLC, have further slowed therapeutic innovation.

The absence of effective early-detection methods further hinders timely intervention [91,92,93,94]. High relapse rates, clonal evolution under therapy, and the emergence of resistant subclones reflect the disease’s genomic instability and impaired DNA damage repair pathways [95]. Consequently, standard therapies frequently fail to produce durable responses, highlighting the urgent need for predictive tools that can detect resistance before clinical progression.

SCLC lacks effective early-detection strategies, though methods such as cfDNA methylation profiling show promise [65]. Standard treatment includes chemoradiation for limited-stage disease and platinum–etoposide plus immunotherapy for extensive-stage disease [76]. Tailoring these therapies to specific molecular subtypes could improve outcomes, but current methods lack sufficient resolution to make such stratification routine. Subtype-specific therapeutic opportunities, for example, AKIs for *MYC*-amplified SCLC-N or ADCs for SCLC-P, could be more precisely matched to patients using AI-driven molecular classification. Other challenges involving subtyping include a lack of SCLC cases, which is one of the most common subtypes and an overlap in CT features which complicates getting accurate diagnoses [38,39].

A multi-omics, nucleic-acid-driven strategy is particularly well-suited to address these gaps by inferring tumor phylogenies and evolutionary trajectories, detecting emerging resistant subclones, predicting relapse earlier, accommodating mixed subtype profiles or lineage switching through integrated multi-model AI biomarker analysis, and reconstructing “virtual biopsies” from radiomics to compensate for scarce tissue. AI can effectively simulate tumor evolution, providing actionable insights into which clones are likely to dominate, and which therapies might forestall resistance.

## 10. Discussion

SCLC represents one of the most aggressive and complex solid tumors, shaped by nucleic-acid-level events, profound subtype plasticity, and multi-layered resistance. Despite initial responses, SCLC treatments fail due to rapid chemoresistance and near-universal relapse [40,47]. The combination of late clinical presentations, high mutation burden, and dynamic phenotypic shifts limits the effectiveness of conventional therapeutic strategies. The older age distribution (60–70 years) [40,64,96] complicates treatment tolerance, while late presentation limits therapeutic windows. Together with the absence of high-resolution early-detection biomarkers, these factors contribute to persistently poor outcomes.

Necessary research and treatment for SCLC urgently require reliable biomarkers for early detection, transformation monitoring, and treatment selection. In addition, a recent study used signaling entropy for survival analysis (SEPA) to distinguish cancer patients with different outcomes, identifying pathway features and integrating that information to improve clinical outcome predictions [54]. Integrating genomics, transcriptomics, epigenomics, proteomics, radiomics, and clinical features through AI-driven analytics is essential to identify resistance mechanisms, personalize therapy, and improve outcomes for this historically recalcitrant malignancy. Our findings indicate that a systematic, multi-omics approach paired with AI can uncover early signs of resistance and transformation that were previously undetectable, paving the way for truly precision-guided interventions. Because most existing AI models are trained on retrospective data and small SCLC-specific cohorts, real-world applicability remains limited and requires prospective validation. This framework can be operationalized through (i) multi-cohort, multi-omics data aggregation and harmonization; (ii) model development with appropriate training and validation strategies, such as cross-validation, which focuses on evaluating the model performance and predictability [97], and external cohorts; (iii) external validation and assessment of clinical utility; and (iv) integration into clinical workflows for decision support. Integrating AI/ML models into clinical environments includes various steps such as identifying clinical scenarios, establishing data infrastructure, creating ML operations, actual clinical workflow integration, continuous engagement and monitoring, interpretability, and outcome measurement. For clinical decisions to be made regarding treatment, the existing clinical decision support frameworks are used when returning the prediction results. The clinicians then use their own expertise and preferences in addition to the AI model’s prediction to create a care pathway document [98]. Future characterization of SCLC molecular subtypes, including plasticity patterns, may enable more effective subtype-specific therapy design, particularly if integrated with high-dimensional AI models. Integration of these molecular insights into clinical practice by fostering novel combination therapies and overcoming the resistance mechanisms that have hindered durable responses will define the future of SCLC management.

AI-enabled, multi-omics integration represents a transformative approach to understanding SCLC. Radiomic and machine-learning studies consistently demonstrate improved survival prediction, response assessment, subtype differentiation, and early detection capabilities in SCLC [29,30,32,33]. While technical challenges such as small sample sizes, scanner variability, lack of standardization, reproducibility concerns, and imaging heterogeneity exist [29,99], the predictive power of AI still exceeds traditional clinical models and provides a roadmap for integrating complex molecular and imaging data into decision-making. Incorporating these AI insights into SCLC research and clinical practice has the potential to advance precision therapy and improve survival.

## 11. Conclusions

The fusion of multi-omics data with AI-driven analysis represents not merely an incremental improvement but a paradigm shift in how SCLC biology, resistance, and therapy are understood. By capturing tumor evolution in silico and predicting treatment response, AI may enable clinicians to anticipate and adapt to disease progression, potentially converting a historically intractable cancer into a manageable condition through proactive, personalized strategies.

## Figures and Tables

**Table 1:** Methodological Frameworks, Machine Learning Models, and Multi-Omics in SCLC.

AI Method/Tool	Primary Application in SCLC	Key Advantages, Metrics, and Findings	References
Next-Generation Sequencing (NGS) and Multi-Omics	Whole-genome, transcriptomic, and proteomic mapping.	Identifies *TP53* and *RB1* inactivation and high mutation burden.	Rudin et al. [40]; George et al. [41]
Liquid Biopsies (cfDNA/ctDNA/CTCs)	Non-invasive monitoring of somatic variants and methylation patterns.	Detected tumor changes in 94% of limited-stage and 100% of extensive-stage SCLC; cfDNA spikes precede radiological progression.	Mohan et al. [50]
Immunohistochemistry (IHC) and Immune Profiling	Subtype characterization via neuroendocrine and immune landscape markers.	Confirms pure vs. combined SCLC types; distinguishes “cold” vs. “hot” tumors.	Gay et al. [46]; Peifer et al. [47]; Travis et al. [51]
AI-Based CT Radiomics	Extracting structural features from contrast and noncontrast CT scans.	Discriminates SCLC from NSCLC and identifies SCLC subtypes; peritumoral features assist in surgical planning.	Shah et al. [36]; Zhou et al. [37]; Lin et al. [38]; Varyukhina et al. [39]
XGBoost Classifier and ML Survival Models	Predicting 1–2-year survival, 90-day mortality, and molecular subtypes.	Achieved the highest precision for early mortality rate; classified molecular subtypes with 96.6% accuracy.	Liang et al. [49]; Heeke et al. [52]
Combined Radiomic-Clinical Models	Multimodal integration of imaging and clinical variables.	Predicted extensive-stage survival (AUC = 0.8487) and limited-stage outcomes (AUC = 0.72/0.71).	Wu et al. [29]; Wang et al. [32]
Deep Learning Models	Automated imaging pattern recognition without manual feature engineering.	Validated 15,000 NLST participants; predicts future lung cancer risk using a single low-dose CT scan.	Mikhael et al. [53]
Signaling Entropy for Survival Analysis (SEPA)	Pathway-level data integration for clinical outcome projection.	Forecasts divergent survival outcomes by quantifying signaling network entropy.	Li et al. [54]

## Data Availability

This article is a review and did not involve the generation or analysis of new datasets. All information discussed derives from previously published sources.

## Supplementary Materials

The following supporting information can be downloaded at: https://www.mdpi.com/article/10.3390/cancers18193159/s1, Table S1: Methodological Frameworks, Machine Learning Models, and Multi-Omics in SCLC.

## Abbreviations

The following abbreviations are used in this manuscript:

ADC	Antibody-Drug Conjugate
AI	Artificial Intelligence
AKI	Aurora Kinase Inhibitor
AI/ML	Artificial Intelligence / Machine Learning
ASCL1	Achaete-Scute Family BHLH Transcription Factor 1
AUC	Area Under the Receiver Operating Characteristic Curve
BCL2	B-Cell Lymphoma 2
BiTE	Bispecific T-Cell Engager
BTK	Bruton Tyrosine Kinase
CHK1	Checkpoint Kinase 1
C-Index	Concordance Index
CT	Computed Tomography
cfDNA	Circulating-Free DNA
ctDNA	Circulating-Tumor DNA
DNA	Deoxyribonucleic Acid
EGFR	Epidermal Growth Factor Receptor
FDA	Food and Drug Administration
GBM	Gradient Boosting Machine
GPCR	G Protein-Coupled Receptor
HER2	Human Epidermal Growth Factor Receptor 2
HR	Hazard Ratio
ISG^+^	Interferon-Stimulated Gene-Positive
KDR	Kinase Insert Domain Receptor (VEGFR2)
KNN	K_Nearest Neighbors
LDCT	Low-Dose Computed Tomography
LUAD	Lung Adenocarcinoma
ML	Machine Learning
MLP	Multilayer Perceptron
MET	Mesenchymal-Epithelial Transition Factor
MHC	Major Histocompatibility Complex
MYC	MYC Proto-Oncogene, BHLH Transcription Factor
NCAM1	Neural Cell Adhesion Molecule 1
NE	Neuroendocrine
NEUROD1	Neuronal Differentiation 1
NLST	National Lung Screening Trial
NMF	Non-Negative Matrix Factorization
NSCLC	Non-Small Cell Lung Cancer
OS	Overall Survival
PARP	Poly (ADP-Ribose) Polymerase
PFS	Progression Free Survival
PD-1	Programmed Cell Death Protein 1
PD-L1	Programmed Death Ligand 1
POU2F3	POU Class 2 Homeobox 3
RB1	Retinoblastoma 1
RF	Random Forest
RNA	Ribonucleic Acid
Rucaparib	Poly ADP-Ribose Polymerase Inhibitor (drug)
SCLC	Small-Cell Lung Cancer
SCLC-A	ASCL1-Driven SCLC Subtype
SCLC-N	NEUROD1-Driven SCLC Subtype
SCLC-P	POU2F3-Driven SCLC Subtype
SCLC-I	Immune-Inflamed SCLC Subtype
SEPA	Signaling Entropy for Survival Analysis
SOX2	SRY-Box Transcription Factor 2
STK11	Serine/Threonine Kinase 11
Sybil	Deep-Learning Lung Cancer Risk Prediction Model
TET2	Tet Methylcytosine Dioxygenase 2
TF	Transcription Factor
TIME	Tumor Immune Microenvironment
TKI	Tyrosine Kinase Inhibitor
TME	Tumor Microenvironment
TP53	Tumor Protein P53
T-SCLC	Transformed Small-Cell Lung Cancer
XGBoost	Extreme Gradient Boosting
